# Single-dose Intravitreal Bevacizumab after Complete Panretinal Photocoagulation in Proliferative Diabetic Retinopathy: an Effective Adjunctive Treatment

**Published:** 2017

**Authors:** Alireza DEHGHANI, Heshmatollah GHANBARI, Abdolsamad MAHDIZADEH, Mohsen POURAZIZI

**Affiliations:** 1 Isfahan Eye Research Center, Department of Ophthalmology, Isfahan University of Medical Sciences, Isfahan, Iran

**Keywords:** Bevacizumab, Vascular Endothelial Growth Factor, Panretinal Photocoagulation, Diabetic Retinopathy, Fluorescein Angiography

## Abstract

Patients with proliferative diabetic retinopathy (PDR) who are refractory to complete panretinal photocoagulation (PRP) have a high risk of severe vision loss. The aim of this study was to evaluate the effectiveness of single-dose intravitreal bevacizumab (IVB) after complete PRP in patients with refractory PDR. Patients with retinal neovascularization (NV) secondary to diabetes mellitus and refractory to complete PRP were enrolled in this study. All patients received a single dose of 1.25 mg IVB at 3 months after completing the PRP. Patients underwent complete ophthalmic evaluation and fluorescein angiography (FA) at baseline and 1 month after injection. The main outcome measure was a reduction in the areas of leakage (mm^2^) on FA. All patients were evaluated at baseline and on every visit at 1 day, 1 week, and 1 month after the injection. A total of 21 consecutive patients (32 eyes) with PDR completed this study. Thirteen (61.9%) patients were female. The mean ± standard deviation (SD) age was 64.1 ± 5.6 years. Complete and partial response of angiographic leakage of NV was noted in 7 (21.9%) and 18 (56.2%) of 32 eyes after a single IVB injection, respectively. No satisfactory response of retinal NV was observed in 7 eyes (21.9%) at 1 month after the injection. No significant ocular or systemic adverse events were observed. A single-dose of IVB could be associated with a satisfactory response of retinal NV, secondary to PDR, in patients who are refractory to complete PRP.

## INTRODUCTION

Diabetic retinopathy (DR) is characterized by the progressive development of well-defined morphological abnormalities in the retinal microvasculature [1, 2]. Proliferative diabetic retinopathy (PDR) is characterized by the formation of new retinal vessels, which may lead to severe vision loss. PDR occurs in response to the ischemia-mediated release of vascular endothelial growth factor (VEGF) into the vitreous cavity [3-5]. Although the current gold standard for the treatment of PDR is panretinal photocoagulation (PRP), not all patients have a complete response and, sometimes, the disease recurs [6, 7]. Patients with PDR who had a complete PRP procedure that did not result in regression or disruption of retinal new vessels have a high risk of severe vision loss [4, 7]. In cases of persistent new vessels in spite of complete PRP, there is a risk of complications such as intravitreal hemorrhage. Because no evidence-based therapy is available for such complicated cases, vitrectomy is often the only option, which by itself increases the risk of an inflammatory and proliferative exacerbation [8, 9]. Intravitreal injections of several drugs in combination with PRP have been shown to achieve more favorable therapeutic outcomes than PRP alone, but none of these agents have been able to substitute the remarkable durability and effectiveness of PRP in preventing vision loss in PDR [8, 10]. One such drug is bevacizumab (Avastin®; Genentech Inc., San Francisco, CA, USA), a recombinant humanized monoclonal antibody that blocks angiogenesis by inhibiting VEGF-A [7, 11, 12]. Some studies showed that VEGF seems to be the major stimulus responsible for an increase in vasopermeability, cell proliferation, and angiogenesis in diabetic retinopathy [8, 13]. Bevacizumab was approved by the U.S. Food and Drug Administration (FDA) for certain metastatic cancers such as lung, breast, renal, and brain cancers [11]. Although not currently approved by the FDA for PDR, the injection of 1.25–2.5 mg of bevacizumab into the vitreous cavity has been performed without significant intraocular toxicity [14].

On the basis of data showing increased levels of VEGF in the vitreous cavity in patients with proliferative retinal diseases, new therapeutic strategies were designed. Nowadays, there are several clinical trials that provided evidence that intravitreally administered anti-VEGF drugs may induce a short-term regression of new vessels in vasoproliferative disease [7, 8, 12, 15, 16]. In this study, we evaluated the effect of single-dose intravitreal bevacizumab (IVB) in patients with refractory PDR after complete PRP.

## MATERIALS AND METHODS

This prospective, uncontrolled, non-randomized clinical study included 32 eyes of 21 patients. The study was conducted at our outpatient department (Diabetic Retinopathy, Department of Ophthalmology, Medical University of Isfahan) at Feiz University Hospital, a tertiary ophthalmology referral center, Isfahan, Iran. The study protocol was approved by the institutional ethical committee of Isfahan University of Medical Sciences, Isfahan, Iran. All participants were informed in detail about the nature of off-label use of this medication and the possible risks. Informed consent was obtained from each participant. Patients aged ≥25 years with refractory PDR and visual acuity of at least 20/200 were enrolled in the study. Refractory PDR in our study was defined as persistent active NV despite complete PRP (minimum of 1200 laser spots) at 3 months or more after the PRP treatment had been completed [17]. The exclusion criteria were as follows: patients with a single eye; history of glaucoma; active ocular inflammation; history of prior vitreoretinal surgery; intraocular surgery (cataract, capsulotomy) within the last 3 months; severe lens opacity precluding fundus examination; known coagulation abnormalities or current use of anticoagulant medications other than aspirin; known allergies to any relevant drugs being used in this study; history of thromboembolic events including myocardial infarction or cerebral vascular accident; uncontrolled hypertension; evidence of external ocular infection; and pregnant or lactating female patients.

**Intravitreal Injections Protocol**

The intravitreal injection of bevacizumab (Avastin®) was performed under sterile conditions using topical tetracaine 0.5% (Anestocaine®, Sina Darou, Tehran, Iran), after which 1.25 mg (0.05 ml) of Avastin® were injected at 4 mm from the limbus using a 30-gauge needle. Immediately after the injection was completed, hand motion and finger counting were evaluated. Postoperatively, patients were instructed to use ciprofloxacin eye drops four times per day for 5 days.

Slit-lamp biomicroscopic examination included IOP (Goldman tonometry); dilated fundus examination was performed at baseline and on every visit at 1 day, 1 week, and 1 month after the injection. Before IVB, standard fluorescein angiography (FA) (Heidelberg Engineering, Heidelberg, Germany) was performed in all patients to demonstrate retinal neovascularization at baseline (3 months after the laser treatment). FA was performed at 1 month after injection. Patients were scheduled for follow-up examination at 1 day, 1 week, and 1 month after the injection. FA images were taken at time points between minute 2 and 3 after fluorescein injection. The areas of leakage were used to evaluate the effects of IVB. The main outcome measure was a reduction in the areas of leakage (mm^2^) on FA [18]. We defined a reduction in the areas of leakage of less than 30% as a no-satisfactory response, from 30% to 80% as a partial response, and of more than 80% as a complete response. Possible ocular and systemic side effects of IVB, including changes in the IOP, were recorded on every visit.

Statistical analysis of the data was performed using SPSS software (version 16.0 SPSS, Inc., Chicago, IL). The Fisher’s exact test and independent samples t-test were used to describe data. A P-value <0.05 was considered statistically significant.

## RESULTS

A total of 21 consecutive patients (32 eyes) with PDR completed this study. PRP had been previously performed on all patients. Of the 21 patients, 13 (61.9%) were female. The mean age was 64.1 ± 5.6 years (age range: 55–75 years). Eight (38.8%) patients had a history of hypertension and 17 (81%) had type II diabetes mellitus. The detailed data and clinical outcomes of patients after IVB are presented in Table 1.

**Table 1:** Clinical Outcomes after Intravitreal Injection of Bevacizumab

	Complete response	Partial response	No satisfactory response
Sex			
** Male (n = 19)**	3 (15.8)	12 (63.1)	4 (21.1)
** Female (n = 13)**	4 (30.8)	6 (46.2)	3 (23.1)
Diabetes			
** IDDM (n = 7)**	0 (0)	6 (85.7)	1 (14.3)
** NIDDM (n = 25)**	7 (28.0)	12 (48.0)	6 (24.0)
Age	63.9 ± 4.6	61.1 ± 4.1	67.7 ± 5.9

IDDM: Insulin-dependent diabetes mellitus; NIDDM: Non-insulin-dependent diabetes mellitus; SD: standard deviation

Data are presented as mean ± SD or No (%).

**Figure 1:**
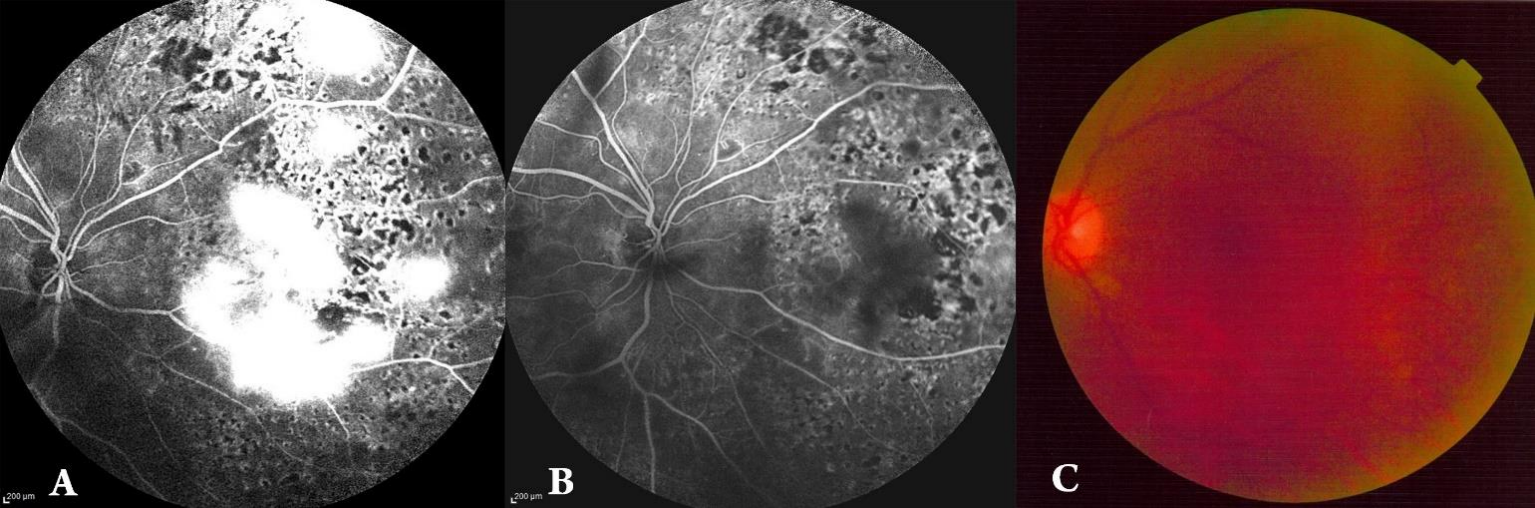
Changes in Amount of Leakage after Intravitreal Bevacizumab Injection in a 55-year-old Man

A: Persistent Active Retinal Neovascularization-associated Diabetes Mellitus in Standard Fluorescein Angiography (Areas of Leakage) despite Complete Panretinal Photocoagulation at 3 Months after the Treatment

B: Reduction the Areas of Leakage (>80%) Found in the Standard Fluorescein Angiography after Single-dose Intravitreal Bevacizumab (Complete Response).

C: Fundus Photograph of the same Patient after Single-dose of Intravitreal Bevacizumab.

At the 1-month follow-up visit after IVB, 21.9% of the treated eyes showed complete response of retinal NV. No satisfactory response of retinal NV was observed in 7 (21.9%) eyes 1 month after the injection. There was no significant different between sex and regression of retinal NV (P = 0.99 by Fisher’s exact test) and type of diabetes and regression of retinal NV (P = 0.99 by Fisher’s exact test). There was a significant difference in age between satisfactory groups (partial and complete groups) versus the no-satisfactory group (61.9 ± 4.4 years versus 67.7 ± 5.9 years, respectively; P = 0.007 by independent samples t-test). No systemic or local adverse events were observed after IVB. No procedure-related complications (e.g., endophthalmitis, uveitis, or ocular toxicity) were observed.

## DISCUSSION

Our study demonstrated that a single-dose IVB could be a promising adjuvant in the treatment of refractory PDR after complete PRP. Since the currently used treatment modality of PRP has its limitations, combined anti-VEGF (Avastin®) therapy in PRP resistance subjects appears as an alternative or adjunctive therapeutic option for PDR. The potential complications of PRP include inevitable devastation of the healthy retina lying next to the photocoagulated area, unfortunate scotomas due to lateral dissipation, and great pain due vertical dissipation [17]. The healthy human retina contains little VEGF; however, patients with active PDR present elevated vitreous levels of VEGF [5]. Increased VEGF, triggered by hypoxia, is a key mediator of retinal NV and macular edema [5, 8, 19]. In pathological processes, the increase in VEGF results in a stimulus to vasodilation, an increase of vascular permeability, endothelial cell proliferation, and activation of metalloproteinases that lyse the extracellular matrix, leaving space for the growth of a new vessel. In PDR, VEGF acts synergistically with other growth factors but is a necessary and sufficient factor for the induction of retinal and iris neovascularization in experimental models [9, 20]. Therefore, inhibition of VEGF by IVB could theoretically provide a potential therapeutic advantage for NV in PDR. The effect of antiangiogenic anti-VEGF on PDR has been studied by several investigators [15, 21]. PRP is a destructive procedure, often painful, and may be associated with a decreased peripheral visual field and an increased risk of macular edema [9, 22]. The current study demonstrated the advantage of a single-dose IVB on the management of patients with PDR after PRP. Some authors have reported reperfused NV after the first IVB [15, 23], a phenomenon that may be attributed to an insufficient amount of bevacizumab. Arevalo et al. reported a dose-dependent response in NV regression in PDR [24]. Although the reason for this dose-dependent response of the retinal neovasculature is unknown and the optimal dose and dosing sequence for IVB is still undetermined, Arevalo et al. demonstrated the 2.5 mg seems to be more effective than the 1.25 mg dose to induce complete NV regression [24]. The clinical explanations are that the effect of IVB on the retinal neovasculature may be affected with other pathologies such as choroidal neovascularization or macular edema or by a previous treatment. However, the real cause remains unknown. Yang et al. hypothesized that the factors influencing the recurrence of retinal NV after the first injection may include the larger area of the ischemic retina, absence of prior laser PRP, staging of preexisting retinal new vessels, and the inadequacy of subsequent laser PRP [25]. This finding was compatible with our study, considering ischemic status may contribute to treatment response. Therefore, a complete PRP may be necessary to sustain a steady outcome of a single dose of IVB in patients with resistant PDR. Previous studies have investigated the benefits of the combination of IVB and PRP [18, 26, 27]. In patients with PDR, the total area of actively leaking NV was significantly reduced in the PRP + IVB group compared with PRP alone [18]. Compatible with our study, Tonello et al. showed the adjuvant use of IVB in PRP for patients with high-risk PDR had a positive effect on the reduction of dye leakage in FA within a short period of time [18]. Although the exact mechanism is unknown, it may be due to an additional effect of IVB in the prevention of leakage and its associated vision-threatening complications. Mason et al. observed a single IVB before standard PRP may be beneficial in preventing PRP-induced visual dysfunction [27]. In contrast to our study, Mason et al. used IVB before PRP. Nevertheless, they showed that combination of IVB and PRP had more benefits than PRP alone since none of the eyes in the combination of IVB and PRP had either worse vision or a significant increase in foveal thickness [27]. Consistent with a previous study, Cho et al. showed the adjuvant use of a single 1.25 mg/0.05 ml IVB in PRP for PDR had a significantly lower occurrence of complications [26]. Schmidinger et al. showed that repeated IVB is a promising method to control retinal new vessels in patients with refractory to PRP. In their study, the leakage area after repeated 1 mg IVB after panretinal PRP during the 6-month follow-up was significantly reduced [28]. Although the dose of 1.0 mg bevacizumab used in the study of Schmidinger et al. was lower than that used in our study, the extent of vessel regression was approximately the same [28]. This may be due to the multiple injections in their study versus a single injection in our study. 

The limitations of the present study include the fact that it had relatively small number of patients (yet sufficient for statistical purposes) and a short-term follow-up period. There was no control group of PDR refractory to PRP without IVB treatment and a long-term prospective study is needed to confirm the maintenance of therapeutic benefit suggested in this study. With respect to limitations, the importance of our study lies in observing the clinical effect of single-dose bevacizumab in the treatment of refractory PDR after complete PRP. Therefore, based on these findings, single-dose bevacizumab may be a promising, safe, and effective adjuvant treatment for refractory PDR after complete PRP. 

In conclusion, the short-term results of our study suggest that single-dose IVB is associated with a satisfactory response of retinal NV secondary to PDR in patients who are refractory to complete PRP. Although this treatment is unlikely to result in the permanent reduction of new retinal vessels, it might be a useful strategy for refractory cases.
